# The complete chloroplast genome sequence of the medicinal plant, *Dracocephalum rupestre* (Lamiaceae)

**DOI:** 10.1080/23802359.2023.2172970

**Published:** 2023-02-02

**Authors:** Eun-Kyeong Han, Tae-Im Heo, Gantsetseg Amarsanaa, Jong-Won Park, Jung-Hyun Lee

**Affiliations:** a Department of Biology Education, Chonnam National University, Gwangju, Republic of Korea; b Baekdudaegan Biodiversity Conservation Division, Baekdudaegan National Arboretum, Bonghwa, Republic of Korea

**Keywords:** Chloroplast genome, tribe Mentheae, *Dracocephalum rupestre*, phylogenetic analysis

## Abstract

*Dracocephalum rupestre* (tribe Mentheae; Lamiaceae) is a perennial herb from Korea and China with high ornamental and medicinal value. Here, we report its complete chloroplast genome to provide insight into the phylogenetic relationships of *Dracocephalum*. The genome is 151,230 bp long, with two inverted repeat regions (25,643 bp each) that separate a large single-copy region (82,536 bp) and a small single-copy region (17,408 bp). It contains 133 genes that encode 88 proteins, eight rRNAs, and 37 tRNAs. The maximum-likelihood phylogenetic analysis strongly supported *Dracocephalum* monophyly, showing that the genus forms a sister group with the subtribe Menthinae in the tribe Mentheae.

## Introduction

The perennial herb *Dracocephalum rupestre* (Hance 1869) belongs to the economically important tribe Mentheae (Lamiaceae). Distributed throughout northern China and Korea (Ren et al. 2008; Drew and Sytsma 2012; Kim et al. 2021), *D. rupestre* has large, fragrant purple-blue flowers that are used for ornamentation (Li and Hedge 1994) and consumed as a tea in Hebei and Shanxi provinces (also known as Maojian tea; Gao et al. 2022). In addition, the herb is an ingredient in traditional Chinese medicine with documented pharmacological benefits, including antioxidant and antitumor activity, as well as liver protection. It is mainly used to treat damp heat, headache, fever, jaundice, and liver toxicity (Esmaeili et al. 2014; Zhu et al. 2018; Gao et al. 2022).

Despite their considerable economic value, little is known about the genetic information of *D. rupestre*. Moreover, the plants are rare in Korea and listed as protected species by the Korea Forest Service (Korea National Arboretum 2008). Populations of Korean *D. rupestre* are restricted to small areas at the southern limit of their geographical distribution (Song et al. 2016; Kim et al. 2018). Here, we report the complete chloroplast genome of *D. rupestre* and examine its phylogenetic position within the tribe Mentheae. The chloroplast genome provides basic genetic data for future phylogenetic and conservation studies.

## Materials and methods

**Figure 1.**
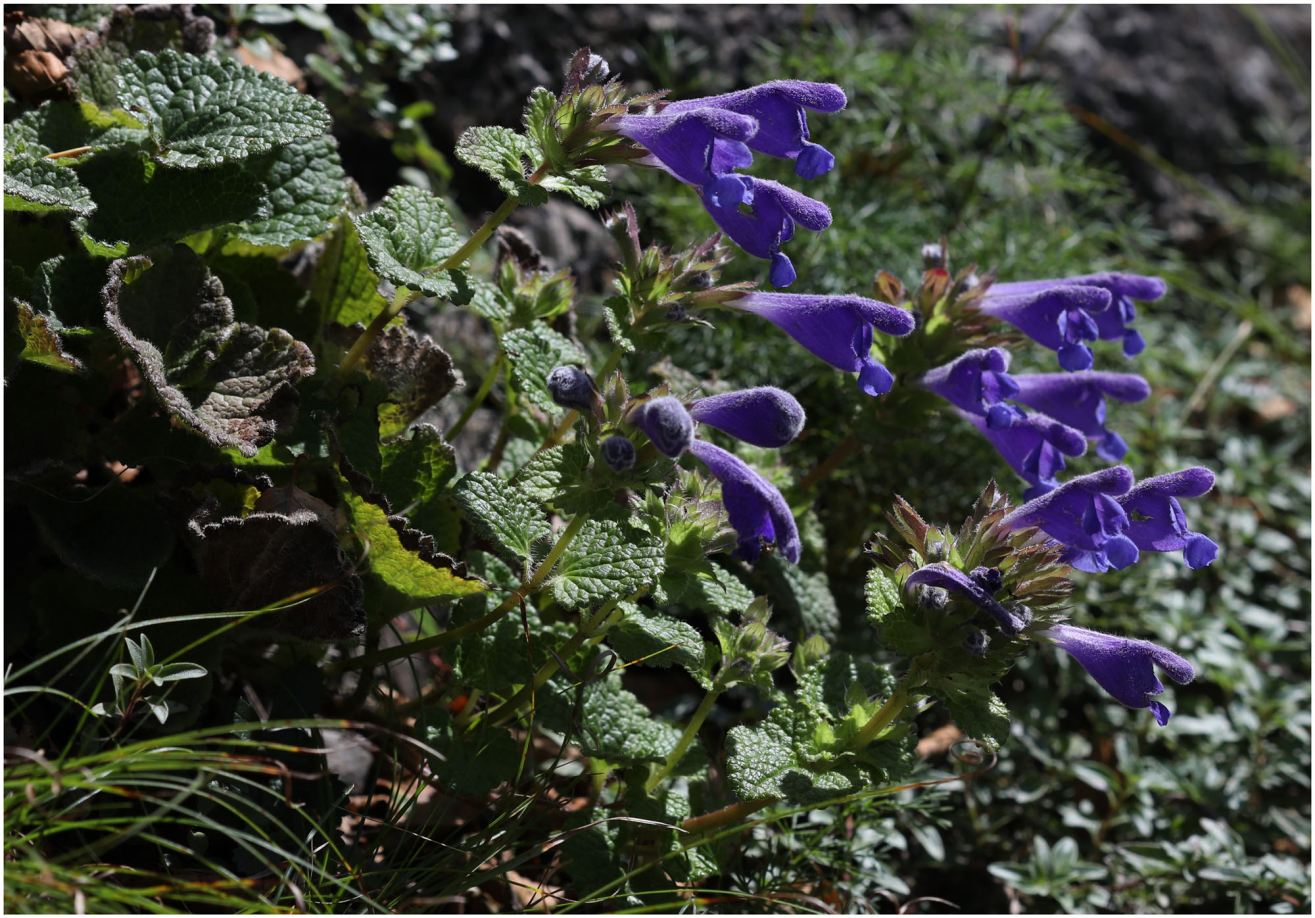
The morphological characteristics of *Dracocephalum rupestre. D. rupestre* is a perennial, 20–30 cm tall. Stems ascending, unbranched, pubescent; leaf blade cordiform or reinform, crenate, white villous; corolla purple-blue, pubescent. The photo was taken by Dr. Tae-Im Heo in Mt. Deokhang, South Korea, June 2022, without any copyright issues.

Fresh leaves of *D. rupestre* were collected from Mt. Deokhang, Gangwon-do, South Korea (37°19′24.13″N, 129°0′17.26ʺE; Figure 1) and dried with silica gel. The voucher specimen (BDNA-SB-20401) was stored in Baekdudaegan National Arboretum (KBA; heoming@koagi.or.kr). Total genomic DNA was extracted from dried leaves using a DNeasy Plant Mini Kit (Qiagen, Seoul, South Korea). The DNA library was constructed with the MGIEasy DNA Library Prep Kit (MGI Technology, Shenzhen, China) and sequenced on the MGISEQ-2000 platform (LAS, Seoul, Korea) following manufacturer protocol. Sequencing generated 75,374,386 raw pair-end reads (150 bp) that were then assembled in NOVOPlasty 4.1 (Dierckxsens et al. 2017). The selected seed sequence was *D. moldavica matK* (Yao et al. 2020; NC_057509). Next, 7,315,587 reads were mapped in Geneious Prime 2022.2.2 (Kearse et al. 2012) to check the assembled plastome, yielding a coverage of 9802× (Figure S1). The plastome was also annotated in Geneious and manually corrected for start and stop codons, as well as for intron/exon boundaries. The complete chloroplast sequence of *D. rupestre* was deposited in GenBank of the National Center for Biotechnology Information (NCBI, accession number OP526971).

For phylogenetic analysis, the complete chloroplast genome sequences of 12 species in Mentheae were downloaded from NCBI (*Dracocephalum*: six species, *Schizonepeta, Nepeta*, *Mentha*, *Prunella*, *Lycopus*, and *Salvia*: one species each). The outgroup was *Lavandula angistifolia* (tribe Lavanduleae). Sequences were aligned in MAFFT (Katoh and Toh 2010). Maximum likelihood (ML) analysis was performed in RAxML v.8.0 (Stamatakis 2014) using default parameters and 1,000 bootstrap replicates. For the RAxML tree, the general time-reversible model of nucleotide substitution was used along with the GTR gamma model of rate heterogeneity.

## Results and discussion

**Figure 2.**
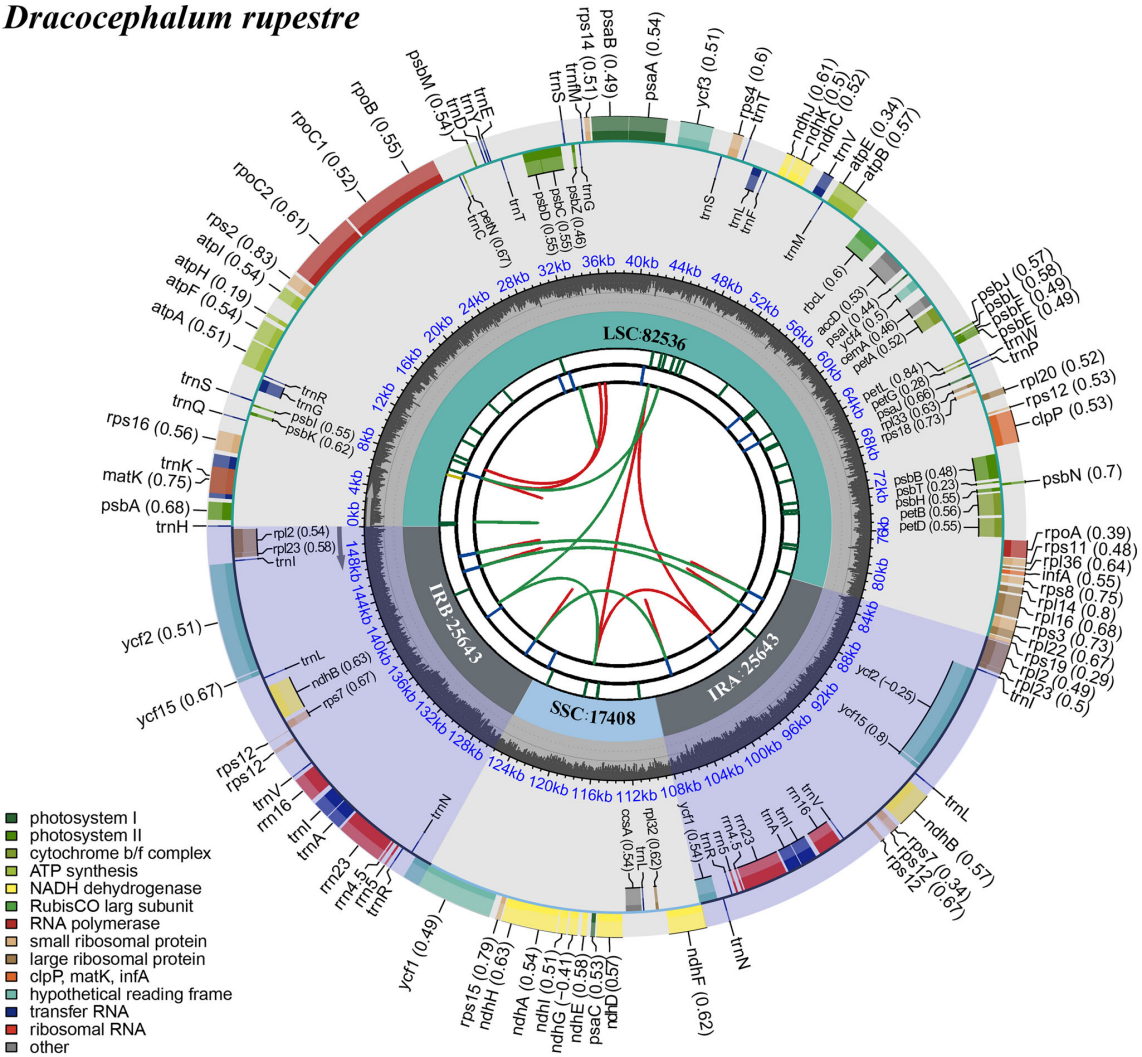
Schematic map of overall features of the chloroplast genome of *Dracocephalum rupestre.* The circular map of the chloroplast genome was generated using CPGview (Liu et al. 2023). The map contains seven circles. From the center going outward, the first circle shows the distributed repeats connected with red (the forward direction) and green (the reverse direction) arcs. The next circle shows the tandem repeats marked with short bars. The third circle shows the microsatellite sequences as short bars. The fourth circle shows the size of the LSC and SSC. The fifth circle shows the IRA and IRB. The sixth circle shows the GC contents along the plastome. The seventh circle shows the genes having different colors based on their functional groups.

The complete chloroplast genome of *D. rupestre* is 151,230 bp and comprises a large single-copy (LSC) region of 82,536 bp, two inverted repeat (IR) regions of 25,643 bp, and a small single-copy (SSC) region of 17,408 bp (Figure 2). We annotated 133 genes, including 88 protein-coding genes (PCGs), eight ribosomal RNA genes (rRNAs), and 37 transfer RNA genes (tRNAs). Of these, 17 were duplicated in IR regions, including six PCGs (*ndhB*, *rpl2*, *rpl23*, *rps7*, *ycf15*, and *ycf2*), four rRNAs (4.5S, 5S, 16S, and 23S rRNA), and seven tRNAs (*trnA-UGC*, *trnI-CAU*, *trnI-GAU*, *trnL-CAA*, *trnN-GUU*, *trnR-ACG*, *and trnV-GAC*). Respectively, *rps19* and *ycf1* were located at the borders of IR/LSC and IR/SSC. Fifteen genes (*trnK-UUU*, *rps16*, *trnG-UCC*, *atpF*, *rpoC1*, *trnL-UAA*, *trnV-UAC*, *petB*, *petD*, *rpl16*, *rpl2*, *ndhB*, *trnI-GAU*, *trnA-UGC*, and *ndhA)* contained one intron, whereas *clpP* and *ycf3* contained two introns. *rps12* is also a trans-spliced gene (Figure S2). Overall GC content of chloroplast DNA was 37.8%, while CG content in the LSC, SSC, and IR regions were 35.9%, 31.7%, and 43.0%, respectively. Gene content and order are similar to those of *D. tanguticum* and *D. heterophyllum* (Yao et al. 2020; Fu et al. 2022).

**Figure 3.**
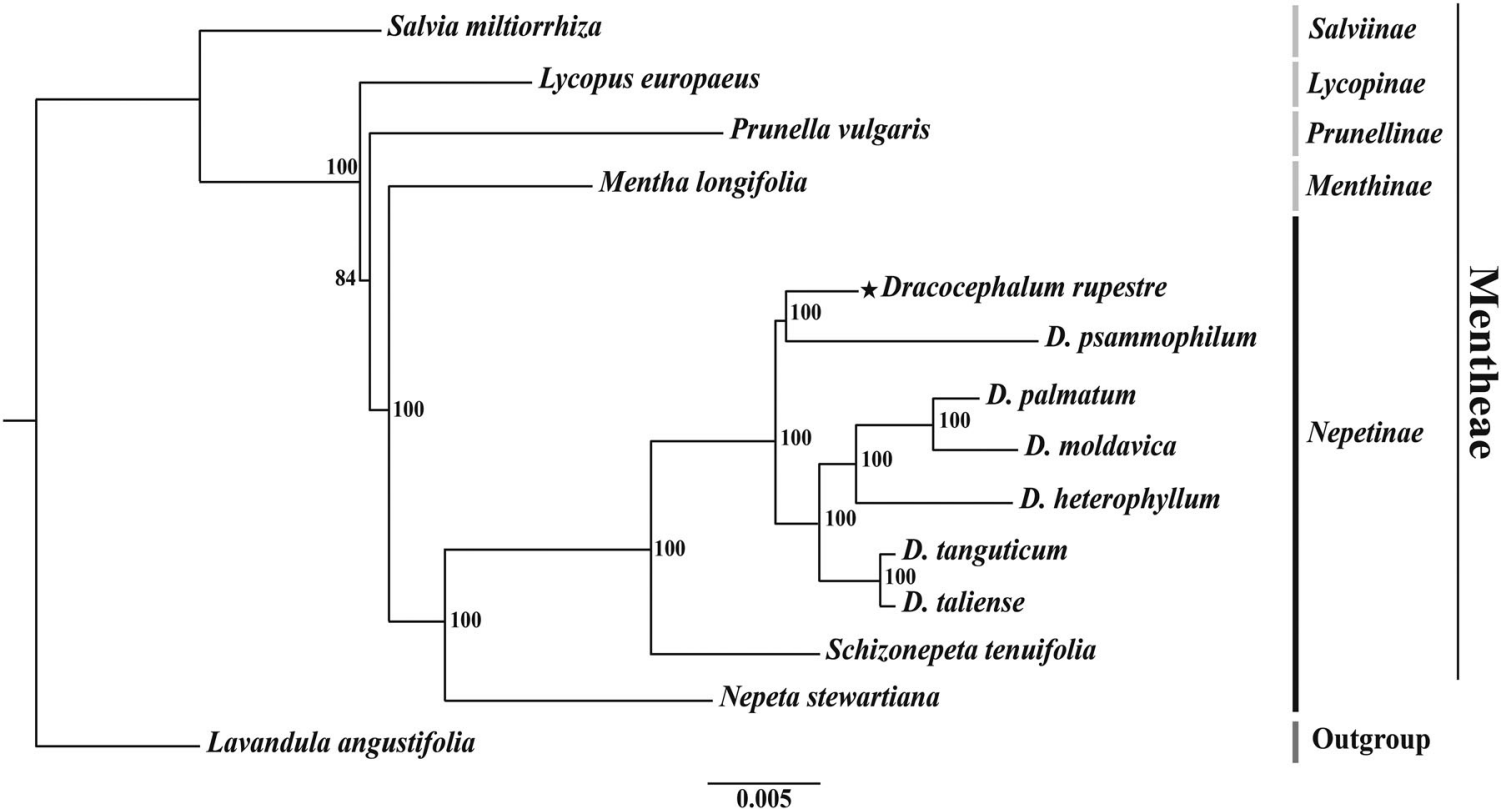
A phylogenetic tree (RAxML) was established based on concatenated sequences of 75 common protein-coding genes in 14 complete chloroplast genomes of the family Lamiaceae. The sequences used for tree construction are as follows: *Salvia miltiorrhiza* (JX312195; Qian et al. 2013), *Lycopus europaeus* (OM617843), *Prunella vulgaris* (MZ636547), *Mentha longifolia* (KU956042; Vining et al. 2017), *Dracocephalum rupestre* (OP526971), *D. psammophilum* (MZ750980), *D. palmatum* (KU958581), *D. moldavica* (MT457747; Yao et al. 2020), *D. heterophyllum* (MW970109; Zhang et al. 2021), *D. tanguticum* (MT457746; Yao et al. 2020), *D. taliense* (MT473756; Zhao et al. 2021), *Schizonepeta tenuifolia* (MW900176), *Nepeta stewartiana* (MT733874; Wu et al. 2021), *Lavandula angustifolia* (KT948988). The numbers above the nodes indicate bootstrap values with 1,000 replicates.

The ML phylogenetic tree showed that *D. rupestre* is closely related to *D. psammophilum*, and all seven *Dracocephalum* species are a monophyletic group with a 100% bootstrap value (Figure 3). Subtribe Nepetinae (comprising *Dracocephalum*, *Schizonepeta*, and *Nepeta*) forms a sister clade to subtribe Menthinae.

In conclusion, this study sequenced the complete chloroplast genome of *D. rupestre*, providing a valuable genetic source for conservation studies. Additionally, the novel phylogenetic data can be used in future evolutionary studies of Lamiaceae.

## Supplementary Material

Supplemental Material

Supplemental Material

Supplemental Material

## Data Availability

Data from this study are available in the NCBI GenBank at https://www.ncbi.nlm.nih.gov (accession number OP526971). The associated BioProject, SRA, and Bio-Sample numbers are PRJNA883819, SRS15224747, and SAMN30996907, respectively.
