# Supplementary figures and images for: The complete chloroplast genome sequence of the medicinal plant, Dracocephalum rupestre (Lamiaceae)
Source: Mitochondrial DNA B Resour. 2023 Feb 2;8(2):229–32. doi: 10.1080/23802359.2023.2172970 (PMC9902028; doi:10.1080/23802359.2023.2172970)

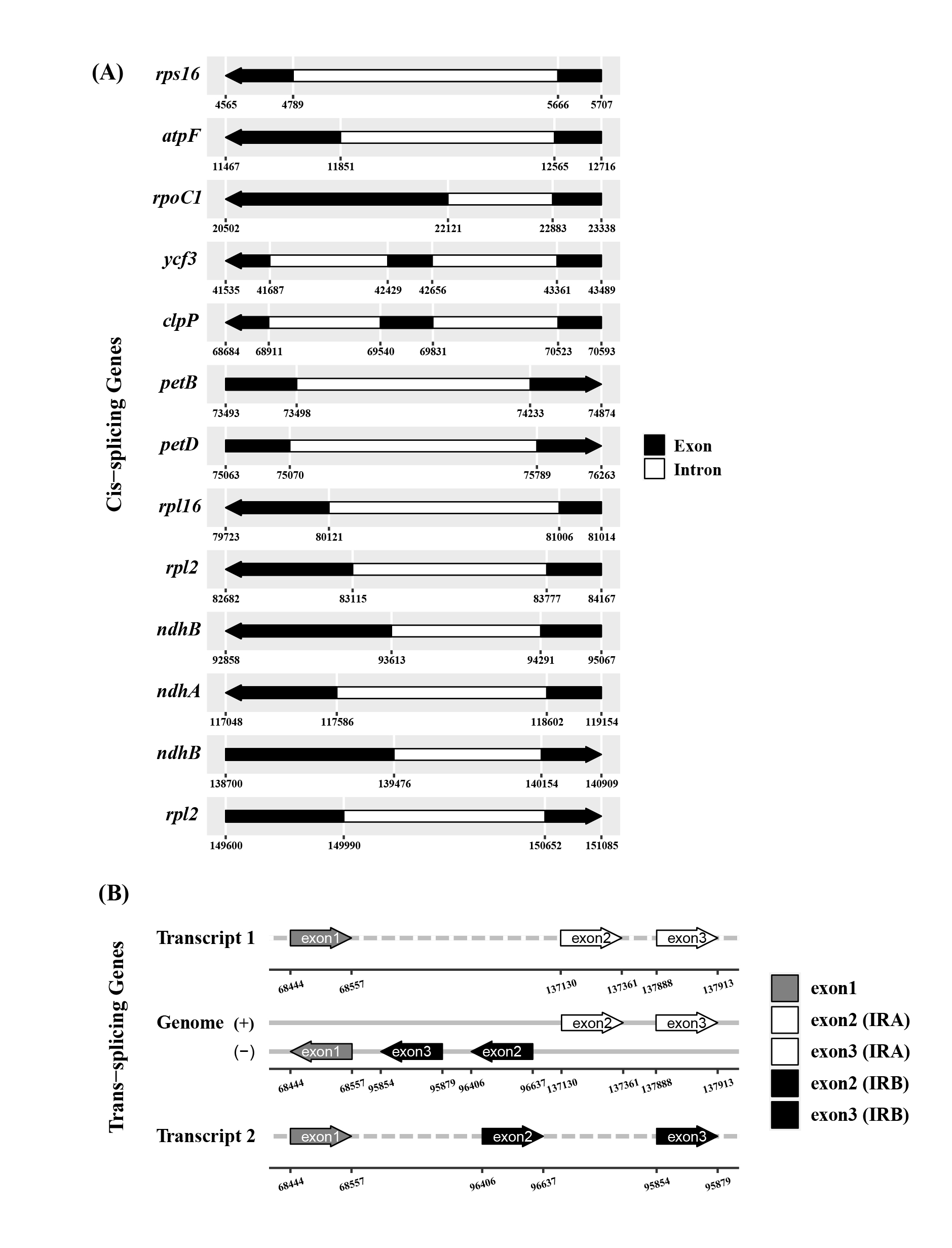

Supplement: Supplemental Material [file TMDN_A_2172970_SM1874.jpg]

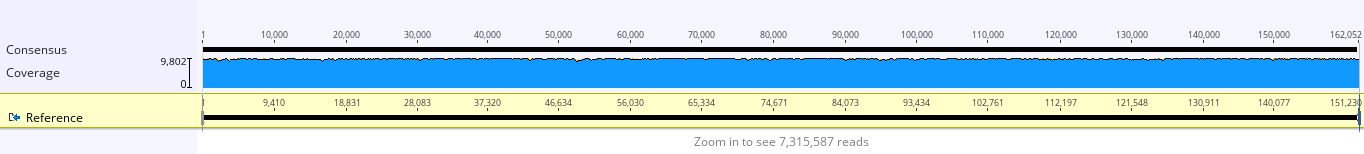

Supplement: Supplemental Material [file TMDN_A_2172970_SM1871.jpg]
