# Supplementary material for: The complete chloroplast genome sequence of the medicinal plant, Dracocephalum rupestre (Lamiaceae)
Source: Mitochondrial DNA B Resour. 2023 Feb 2;8(2):229–32. doi: 10.1080/23802359.2023.2172970 (PMC9902028; doi:10.1080/23802359.2023.2172970)
Supplement: Supplemental Material [file TMDN_A_2172970_SM1869.docx]

Figure captions

Figure S1. Overall coverage depth of the chloroplast genome assembly of *Dracocephalum rupestre*.

Figure S2. Schematic map of the cis and trans splicing genes in the chloroplast genome of *Dracocephalum rupestre*.


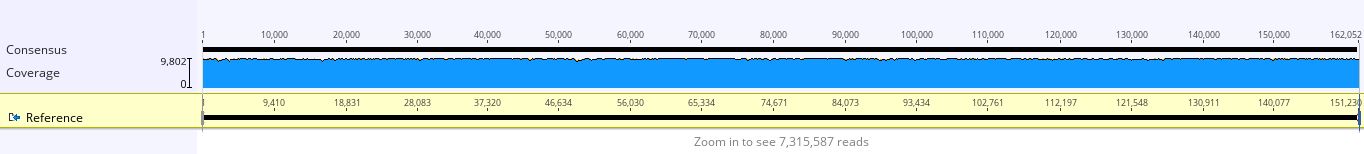


Figure S1. Overall coverage depth of the chloroplast genome assembly of *Dracocephalum rupestre*. This figure was generated using Geneious Prime by aligning DNA-Seq data to the whole chloroplast genome. The height of the blue graph indicates the number of sequences at each location.


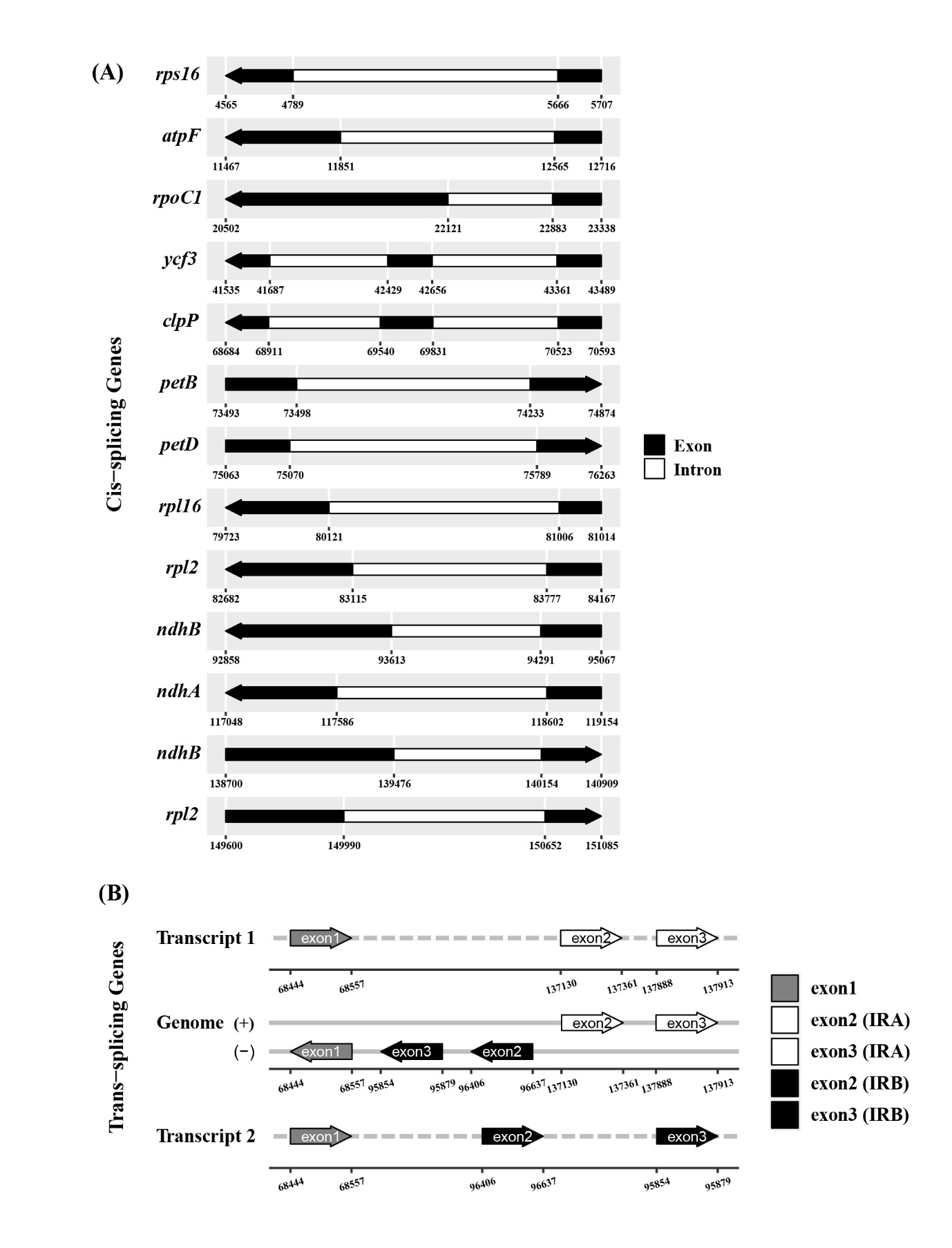


Figure S2. Schematic map of the cis-splicing genes (A) and trans-splicing gene rps12 (B) in the chloroplast genome of *Dracocephalum rupestre*. The exons are shown in black; the introns are shown in white. The arrow indicates the sense direction of the gene. The map was generated using CPGview.
